# The Remineralization Potential of Fluoride, Casein Phosphopeptide-Amorphous Calcium Phosphate, and Chicken Eggshell on Enamel Lesions: An In Vitro Study

**DOI:** 10.7759/cureus.77396

**Published:** 2025-01-13

**Authors:** Siddhesh Bandekar, Shreyash Parkhi, Shirin Kshirsagar, Nikhil R Sathawane, Waseem A Khan, Priyanka Razdan

**Affiliations:** 1 Department of Conservative Dentistry and Endodontics, Yogita Dental College and Hospital, Khed, IND; 2 Department of Conservative Dentistry and Endodontics, Swargiya Dadasaheb Kalmegh Smruti Dental College and Hospital, Nagpur, IND; 3 Department of Paediatric and Preventive Dentistry, Yogita Dental College and Hospital, Khed, IND

**Keywords:** casein phosphopeptide-amorphous calcium phosphate, dental caries, eggshell, fluoride varnish, remineralization

## Abstract

Background and objective

The effectiveness of various remineralizing agents, including fluoride varnish, casein phosphopeptide-amorphous calcium phosphate (CPP-ACP), and chicken eggshell powder (CESP), in improving enamel surface microhardness (SMH) is of clinical interest. This in vitro study aimed to evaluate and compare SMH changes in the enamel after treatment with these agents.

Materials and methods

An in vitro study was conducted in which 40 human premolars extracted for orthodontic reasons were sectioned into four enamel sections, which were randomly allocated into four groups: group A (fluoride varnish), group B (CPP-ACP cream), group C (CESP), and group D (control). Group A was treated with a thin layer of fluoride varnish (MI Varnish®, GC Corp., Tokyo, Japan), which was left to absorb for 20 s before air drying. Group B specimens were treated with CPP-ACP cream (GC Tooth Mousse®; GC Corp.) for at least three minutes. Group C received CESP application to the enamel surface, which was allowed to absorb for five minutes. Group D (the control group) did not receive any surface treatment. Artificial carious lesions were induced using a demineralization solution and pH cycling for five days. The treated samples were stored in artificial saliva for 21 days at 37 °C. The SMH was assessed using a Vickers microhardness tester at baseline, post-demineralization, and post-treatment. Statistical analyses included paired t-test, one-way analysis of variance (ANOVA), and Tukey’s post-hoc test.

Results

After demineralization, SMH significantly decreased in all groups (p<0.05). After treatment with various remineralizing agents, groups A, B, and C demonstrated significant improvement in SMH compared to the control group (p<0.05). Group C exhibited the greatest improvement in SMH, followed by groups A and B. The difference in the SMH between the treatment groups was statistically significant (p<0.05).

Conclusions

All three remineralizing agents effectively increased SMH, with CESP showing superior results. Fluoride varnish and CPP-ACP also demonstrated substantial remineralization potential. Further in vivo studies are required to validate these findings in a clinical setting.

## Introduction

Dental enamel serves as the primary protective shield for teeth and is characterized by its high mineral content, which is essential for safeguarding against mechanical, thermal, and chemical stressors. However, enamel is highly susceptible to demineralization, a process driven by acidic byproducts of bacterial activity, dietary acids, and reduction in salivary pH [1]. These factors disrupt the equilibrium between demineralization and remineralization, often leading to the development of early enamel lesions. Given that enamel lacks regenerative ability due to the absence of cellular repair mechanisms, it is crucial to address demineralization at its earliest stages [2].

Remineralization is the process through which minerals such as calcium (Ca^2+^) and phosphate (PO_4_^3-^) are redeposited into demineralized enamel. Several therapeutic agents with unique mechanisms and advantages have been developed to support this process. Fluoride has long been regarded as the gold standard in preventive dentistry owing to its ability to form fluorapatite, a mineral that is more resistant to acidic environments than hydroxyapatite [3]. Additionally, fluoride promotes the remineralization of enamel by enhancing the absorption of Ca^2+^ and PO_4_^3-^ ions, while reducing enamel solubility [4].

Casein phosphopeptide-amorphous calcium phosphate (CPP-ACP) is another advanced remineralization agent. The CPP component stabilizes ACP in its amorphous state, ensuring that Ca^2+^ and PO_4_^3−^ ions remain bioavailable for targeted delivery to areas of enamel demineralization [5]. This mechanism significantly accelerates the remineralization process, contributing to enamel restoration and reducing the likelihood of further demineralization [6].

Recently, natural and cost-effective remineralization agents such as chicken eggshell powder (CESP) have gained attention. In calcium carbonate and essential trace minerals, CESP is believed to have remineralizing effects similar to those of synthetic agents [7]. Research indicates that Ca^2+^ ions from eggshells interact with PO_4_^3-^ ions in saliva or artificial environments to promote mineral deposition on demineralized enamel surfaces [8,9].

Despite the growing interest in these materials, there is a lack of sufficient data in the literature comparing their efficacy in remineralizing enamel defects. Hence, the current study was conducted as an in vitro investigation to evaluate and compare the remineralization potential of CESP, fluoride varnish, and CPP-ACP in enamel lesions.

## Materials and methods

Study setting

This in vitro study was conducted to assess changes in enamel surface microhardness (SMH) following various remineralization treatments. This study was conducted in a controlled laboratory setting at Yogita Dental College, Khed, within the Department of Conservative Dentistry and Endodontics, from January 2024 to May 2024. The study adhered to the checklist for reporting in vitro studies (CRIS) guidelines for in vitro research and received approval from the institutional ethical committee (approval no: YDCH/IEC/2107/12/2023) [10]. Written consent was obtained from all patients to use their extracted teeth for our study.

Sample size estimation

The sample size was determined using G Power software, version 3.2.9 (Heinrich-Heine-Universität Düsseldorf, Düsseldorf, Germany). To achieve statistically significant differences in the surface hardness among the four treatment groups, a minimum of 40 samples was required. With 80% power and a 5% alpha error, an effect size of 0.55, based on prior studies, was considered [11]. To standardize the sample for each group, ten extracted premolar teeth were collected, with each crown divided into four equal parts for inclusion in the experimental groups.

Inclusion and exclusion criteria

The inclusion criteria for sample selection were as follows: premolar teeth extracted for orthodontic reasons from patients aged 14-20 years with no caries, fluorosis, demineralization, cracks, or restorations. The collected teeth were stored in a 0.1% thymol solution during sample preparation for a period of one month, and the solution was changed weekly to maintain its effectiveness. 

Sample preparation

**Figure 1:**
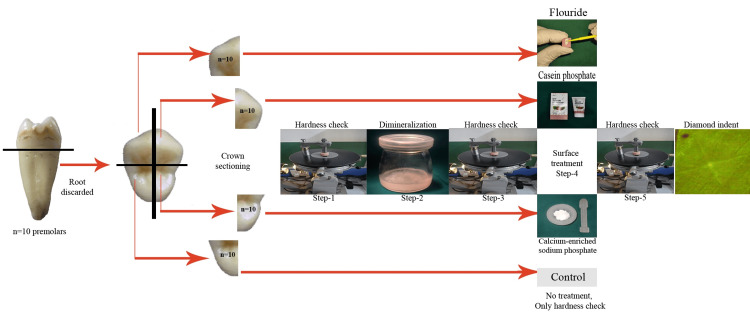
Flowchart of the methodology illustrating the preparation and treatment of extracted premolars

The roots of each tooth were severed at the cemento-enamel junction, and the coronal portion was longitudinally sectioned into four enamel segments (buccolingual and mesiodistal) using a high-speed diamond-tipped disc (IsoMet Blade 5LC; Buehler Inc., Lake Bluff, IL) attached to a precision cutting apparatus (Figure 1).

The enamel surfaces were abraded with silicon carbide abrasive papers (grits 1000-1200, Carborundum Universal Ltd., Tamil Nadu, India) in a wet environment, creating uniform, flat enamel surfaces while preserving the subjacent dentin. Each enamel section was then embedded in custom-made cylindrical molds filled with self-cured acrylic resin, leaving a 3×3 mm window of enamel exposed at the center (Figure 1). These specimens were randomly assigned to one of four experimental groups to evaluate the efficacy of different surface treatments.

Preparation of CESP

CESP was prepared according to the World Intellectual Property Organization guidelines (WO/2004/105912: Method of Producing Eggshell Powder). The calcination process was aimed at increasing the alkalinity of the natural powder and ensuring that it was pathogen-free. Twenty chicken eggs were sourced from a local hatchery, and the eggshells were cleaned with distilled water after removing the egg contents (India Chemicals Pvt. Ltd., Mumbai, India). The inner membrane was carefully removed by exposing the eggshells to a water bath at 100°C for 10 min. The shells were then crushed using a sterilized mortar and pestle, followed by heating in a muffle furnace at 1200°C. After calcination, the powder was milled to a fine consistency [12]. A 1 g sample of CESP was dissolved in 20 mL of 4% acetic acid (India Chemicals Pvt. Ltd., Mumbai, India) to prepare the CESP solution. The clear supernatant was transferred to a beaker, and the pH was measured using a pH meter (Deluxe Deep Vision, Model No. 101, California, USA), which had a pH of 11.7.

Methodology

To simulate demineralization without affecting the surrounding tooth structure, acid-resistant nail polish was uniformly applied to all surfaces, except the window in the experimental groups (groups A, B, and C). Group D served as the positive control. The demineralization solution was prepared by dissolving 2.2 mmol/L of calcium chloride (CaCl₂), 2.2 mmol/L of potassium phosphate (KH₂PO₄), and 0.15 mol/L of sodium chloride (NaCl) in distilled water. The pH was adjusted to 4.5 using 0.1 mol/L acetic acid (Alpha Chemika, Mumbai, India). Samples in groups A, B, and C underwent cyclic pH variations by immersing them in 35.5 mL of demineralization solution per block for 3 h per day, followed by immersion in 17.75 mL of artificial saliva for 21 h, simulating oral conditions for five days to induce artificial carious lesions in the enamel.

Group A was treated with a thin layer of fluoride varnish (MI Varnish®, GC Corp., Tokyo, Japan), which was left to absorb for 20 s before air drying. Group B specimens were treated with CPP-ACP cream (GC Tooth Mousse®; GC Corp., Tokyo, Japan) for at least three minutes. Group C received CESP application to the enamel surface, which was allowed to absorb for five minutes. Group D (the control group) did not receive any surface treatment. All the surface treatments were performed only once. All specimens were then stored in 100 mL of artificial saliva (Biotene, GlaxoSmithKline, London, United Kingdom) at 37°C for 21 days, with saliva replaced every two days. Artificial saliva contains various components such as ions calcium, phosphate, and fluoride ions that can degrade or precipitate over time. Replacing the solution ensured that the chemical composition remained stable, mimicking natural saliva more accurately. To ensure consistency, all the procedures were performed by a single trained and calibrated investigator (Figure 1).

The SMH of enamel was assessed using a Leica Vickers microhardness tester (Leica Microsystems, Wetzlar, Germany). Baseline SMH was measured, followed by assessments after demineralization and after each treatment with remineralizing agents in groups A, B, and C. A load of 25 g was applied for 5 s and the Vickers hardness number (VHN) was calculated from five indentations spaced 100 µm apart. The mean VHN value for each specimen was recorded.

Statistical analysis

Data were analyzed using SPSS Statistics Version 23.0. (IBM Corp., Armonk, NY). Descriptive statistics were presented as mean ± standard deviation (SD). The Shapiro-Wilk test was used to assess data normality, and data was found to be normally distributed. Paired t-test was used to compare the SMH values before and after treatment. One-way analysis of variance (ANOVA) followed by post-hoc Tukey’s test was used for group comparisons. Statistical significance was set at p<0.05.

## Results

Comparison of the mean SMH values at various intervals revealed no statistically significant differences between the groups at baseline and after demineralization (p>0.05), confirming that the groups were comparable without bias. However, significant differences were observed among the groups following the remineralization phase (p<0.05). Across all groups, the mean SMH decreased after demineralization and subsequently increased during remineralization. Among the experimental groups, the highest mean SMH value after remineralization was recorded in group C (258.30 ± 13.22) with CESP, whereas the lowest value was observed in group B (206.70 ± 3.43) with CPP-ACP. The control group showed an increase in SMH after immersion in artificial saliva (Table 1).

**Table 1:** Comparison of mean SMH at different treatment intervals by one-way ANOVA test ^*^Statistically significant SMH presented in VHN units ANOVA: analysis of variance; SD: standard deviation; SMH: surface microhardness; VHN: Vickers hardness number

Groups	N (%)	Baseline		P-value	Demineralization		P-value	Remineralization		P-value
		Mean	SD		Mean	SD		Mean	SD	
Group A	10 (25)	277.90	10.70	0.814	185.90	8.15	0.741	219.60	4.35	0.001^*^
Group B	10 (25)	282.40	14.99		188.10	8.75		206.70	3.43	
Group C	10 (25)	283.40	16.52		184.80	14.32		258.30	13.22	
Group D	10 (25)	281.50	10.77		182.70	10.82		186.50	10.69	

Post-hoc Tukey's analysis performed after the remineralization phase identified significant differences in SMH between most group pairs. Group C exhibited the largest mean difference compared to group D (p=0.001), indicating a notable improvement in SMH with CESP application. The smallest mean difference was found between groups A and B, suggesting that both fluoride varnish and CPP-ACP were effective remineralizing agents (Table 2).

**Table 2:** Post-hoc analysis with Tukey test for pairwise comparison of SMH after remineralization process ^*^Statistically significant SMH presented in VHN units SMH: surface microhardness; VHN: Vickers hardness number

Paired group		Mean difference of SMH	P-value	95% confidence interval	
				Lower bound	Upper bound
Group A	Group B	12.9	0.014^*^	2.13	23.67
Group A	Group C	-38.7	0.001^*^	-49.47	-27.93
Group A	Group D	33.1	0.001^*^	22.33	43.87
Group B	Group C	-51.6	0.001^*^	-62.37	-40.83
Group B	Group D	20.2	0.001*	9.43	30.97
Group C	Group D	71.8	0.001^*^	61.03	82.57

Within-group analysis showed statistically significant reductions in SMH after demineralization across all the groups (p=0.001). Although none of the treatments fully restored the initial SMH values, group C achieved the closest approximation to the original enamel, followed by groups A and B. This highlights the superior efficacy of CESP in promoting remineralization, with the fluoride varnish and CPP-ACP showing moderate effectiveness. Group D exhibited the most substantial decline in SMH, reflecting the effects of demineralization (Table 3).

**Table 3:** Comparison of SMH pre- and post-remineralization by paired t-test ^*^Statistically significant SMH presented in VHN units SD: standard deviation; SMH: surface microhardness; VHN: Vickers hardness number

Groups	N (%)	Pre-treatment		Post-treatment		t value	P-value
		Mean	SD	Mean	SD		
Group A	10 (25)	277.90	10.70	219.60	4.35	15.90	0.001^*^
Group B	10 (25)	282.40	14.98	206.70	3.43	15.72	0.001^*^
Group C	10 (25)	283.40	16.52	258.30	13.22	3.74	0.001^*^
Group D	10 (25)	281.50	10.77	186.50	10.69	19.79	0.001^*^

## Discussion

Microorganisms in bacterial plaques synthesize organic acids, which penetrate the pellicle and reach the enamel surface. These acids interact with apatite crystals, primarily targeting the weak lattice sites where the carbonate ions reside. This interaction results in the release of Ca^2+^, PO_4_^3−^, fluoride, carbonate, sodium, magnesium, and hydroxyl ions from the crystal lattice, which subsequently diffuse into the surrounding environment. The released Ca^2+^ and PO_4_^3-^ contribute to the formation of Ca^2+^PO_4_^3-^ salts, which either migrate outward or facilitate the repair of damaged crystallites beneath the enamel surface, aiding in remineralization. Demineralization persists when the acid supply is sufficient. As enamel dissolution progresses, Ca^2+^ and PO_4_^3-^ ion concentrations increase, which enhances the likelihood of surface remineralization. This leads to the development of an intact surface layer, approximately 20-40 µm thick, with a mineral composition superior to that of the body [13].

In this study, enamel surface alterations were quantitatively analyzed using SMH measurements. Microhardness testing is advantageous for assessing materials such as enamel, which has heterogeneous and complex structures and is prone to fracture. The indentation technique is a straightforward, reliable, and minimally invasive method for monitoring changes in enamel remineralization [14]. Artificial saliva, owing to its similar pH and composition to natural saliva, serves as an optimal medium for preserving samples, maintaining equilibrium in inorganic and organic components, and preventing mineral depletion [15].

CESP, which is rich in Ca^2+^, is commonly used as a dietary supplement, especially in the elderly and postmenopausal women. Calcination enhances alkalinity and removes pathogens, whereas a 4% acetic acid solution ensures a pathogen-free preparation. The high pH of the solution (11.8) increases the activity of hydroxyl and phosphate ions, facilitating enamel remineralization. Conversely, acidic solutions reduce the availability of these ions, impairing remineralization. Prior studies have demonstrated CESP’s potential of CESP in enamel remineralization [16-18]. Fathy Abo-Elmahasen et al. [16] used 20% of CESP and concluded that the CESP had an excellent remineralization effect on the demineralized enamel surface after debonding the orthodontic enamel surface.

In this study, the fluoride varnish contained CPP-ACP along with fluoride ions. Additional fluoride contributed to enhanced remineralization and SMH compared to CPP-ACP alone, which is consistent with the findings of Chandak et al. [19]. CPP-ACP acts as a reservoir for bioavailable Ca^2+^ and PO_4_^3-^, maintaining a supersaturated environment for enamel remineralization. In the presence of fluoride ions, it forms fluorapatite (Ca₅(PO₄)₃F), a mineral more resistant to acid than hydroxyapatite. Fluoride ions also react with Ca^2+^ to produce calcium fluoride (CaF₂) on the enamel surface, providing a slow-release mechanism under acidic conditions to support remineralization and protect against caries [3,20]. Reynold et al. advised the use of 1% CPP-ACP solution, replacing 63.9 ±20.1% of mineral lost at an average rate of 3.9 ± 0.8 x 10-8 mol hydroxyapatite/m2/s [20]. However, Lata et al. [21] found CPP-ACP to be more effective than fluoride when studied over seven days, likely due to methodological differences, as the present study spanned 21 days.

CPP-ACP binds strongly to hydroxyapatite, adhering to the enamel and dentin surfaces, particularly in areas of initial demineralization. Upon adhesion, it releases Ca^2+^ and PO_4_^3-^ ions, replenishing the enamel minerals. It creates reservoirs of these ions on enamel surfaces and within dental plaque, which release ions when the pH drops, mitigating acid damage and preventing further demineralization. By restoring the mineral content, CPP-ACP improves enamel microhardness, making it more resistant to abrasion and acid erosion [5,11,19].

Clinical implications

The combination of CPP-ACP, fluoride, and CESP represents a significant advancement in enamel remineralization strategies. These agents repair early enamel lesions, improve SMH, and prevent further demineralization. CPP-ACP serves as a source of bioavailable Ca^2+^ and PO_4_^3-^, while fluoride strengthens enamel by forming acid-resistant Ca₅(PO₄)₃F. These compounds can be integrated into varnishes, toothpaste, and topical treatments to address dental caries and erosion. Additionally, CESP’s high alkalinity of CESP creates an ideal environment for remineralization, making it a promising adjunct in preventive and restorative dentistry.

Limitations

The controlled laboratory setting used in this study may not accurately replicate the dynamic oral environment, including saliva flow, diet, and microbial activity. The use of extracted teeth and the small sample size may limit the applicability of these findings to clinical scenarios. Moreover, the short study duration did not address the long-term effects of these treatments. The uniformity of artificial lesions may not reflect the variability of natural carious lesions. Variations in agent application and absorption could also affect the outcomes. The high pH is hypothesized to contribute to the efficacy of CESP, and therefore, further studies are needed to confirm this mechanism. We also recommend further in vivo studies to validate these results.

## Conclusions

Within the scope of this in vitro study, CESP demonstrated the highest efficacy in remineralizing early enamel lesions, achieving the highest SMH values among all treatments. This highlights the superior efficacy of CESP in promoting remineralization, with the fluoride varnish and CPP-ACP showing moderate effectiveness. This emphasizes its significant role in dental health interventions. All groups performed better than the control group.
